# Effects of Physical and Chemical Modification of Sunflower Cake on Polyurethane Composite Foam Properties

**DOI:** 10.3390/ma14061414

**Published:** 2021-03-15

**Authors:** Anna Strąkowska, Sylwia Członka, Agnė Kairytė, Krzysztof Strzelec

**Affiliations:** 1 Institute of Polymer & Dye Technology, Lodz University of Technology, 90-924 Lodz, Poland; sylwia.czlonka@dokt.p.lodz.pl (S.C.); krzysztof.strzelec@p.lodz.pl (K.S.); 2 Laboratory of Thermal Insulating Materials and Acoustics, Faculty of Civil Engineering, Institute of Building Materials, Vilnius Gediminas Technical University, LT-08217 Vilnius, Lithuania; agne.kairyte@vgtu.lt

**Keywords:** polyurethane foam, sunflower cake, porous structure, mechanical properties, physical and chemical modification

## Abstract

Sunflower cake (SC), which is waste during the production of sunflower oil, was selected as a modifier of properties in polyurethane (PUR) foams. The SC was chemically modified with triphenylsilanol (SC_S) and physically modified with rapeseed oil (SC_O). The influence of SC on the rheological properties of the polyol and the kinetics of foam growth were investigated. PUR foams were characterized by morphological, mechanical, and thermal analysis. The results show that the physical and chemical modification of SC contributes to the changes in the properties of the foams in different ways. Too high hydrophobicity of SC_O affects the structure deterioration, and thus the mechanical properties, and in turn, reduces the affinity for water. In turn, chemical modification with silane allows for obtaining foams with the best mechanical properties.

## 1. Introduction

Polyurethane (PUR) foams possess great properties, such as a relatively simple forming process, aging resistance, and excellent insulating properties. Due to this, they have found an application in many industries, such as construction, automotive, aerospace, and military [1,2,3,4]. The main disadvantage of these PUR materials is their low thermal stability and relatively poor mechanical properties. It has been shown in previous works that incorporation of inorganic or organic fillers can significantly improve the mechanical and thermal performances of PUR composites [5,6,7,8]. For example, Kurańska et al. [9] analyzed the effect of the addition of basalt powder in rigid PUR foams as a filler. A significant improvement in mechanical characteristics of PUR composites was observed on the addition of 3–40 wt % of the filler. Paciorek-Sadowska et al. [5] enhanced rigid PUR foams with rapeseed cake. A significant increase in apparent density and improvement in mechanical properties of rigid PUR foams were observed after the incorporation of rapeseed cake in the amount of 30–60 wt %. Rigid PUR foams synthesized from rapeseed-based polyol were additionally enhanced with egg shells by Leszczyńska et al. [10]. It was reported that the mechanical strength and thermal performances were improved after the incorporation of 20 wt % of the filler. The effect of incorporation of waste sludge particles (PWS) in PUR composites was studied by Kairyte et al. [11]. Results showed that the incorporation of 20 wt % of PWS increased the apparent density of PUR composites and significantly improved their mechanical performances, such as compressive strength and modulus elasticity.

Sunflower cake (SC) is a by-product of sunflower oil production, which is produced after pressing sunflower seeds [12,13]. It is a lignocellulosic raw material containing components insoluble in water. Due to the large production of sunflower oil in Europe, including Poland, a lot of waste in the form of SC is generated every year. Due to the high content of protein (approx. 28%) and fat (approx. 15%) as well as the balanced composition of amino acids, they are used as a supplementary feed in animal husbandry. However, this proportion must be limited to around 15% in complete feed for fattening pigs due to the high fiber content and the low threonine and lysine content. Due to its calorific value, approx. 19 MJ kg^−1^ SC is used as an energy source. However, it is necessary to increase the energy value by co-firing with products with higher calorific value [14].

Despite such a wide use of SC, new applications of this bio-raw material are sought to avoid its overproduction in the future because a significant amount of SC waste may cause environmental problems, for example in processing plants, mainly due to the low stability of these by-products [12].

It is also known that substitutes for synthetic components used in chemistry and technology of polymeric materials are increasingly being sought in order to produce more and more environmentally friendly materials. Therefore, it is justified to introduce waste biomass that could contribute to the reinforcement of the material, making it slightly more environmentally friendly. Taking into account previous studies, which found that bio-waste fillers can be successfully used as a filler in the production of polymer composites [15,16,17,18,19,20], the use of SC as a lignocellulosic reinforcing filler for the production of a rigid PUR composite seems fully justified.

This study concerns the application of SC on the selected properties of rigid PUR composite foams. Non-treated, as well as chemically treated (silanization) and physically treated (oil impregnation), SC was used in the amount of 3 wt %. The influence of non-treated, SC_S, and SC_O on the cellular morphology, apparent density, mechanical performances, thermal stability, insulating properties, and hydrophobic character of PUR composite foams was examined and discussed.

## 2. Materials and Methods

### 2.1. Materials

The water blown PUR used in this test were obtained from a two-component system. Component A was a polyol premix, the calculated amounts of polyol (STEPANPOL PS-2352 from Stepan Deutschland GMBH, Wesseling, Germany—a modified aromatic polyester polyol), catalysts (Kosmos 75—standard potassium octoate and Kosmos 33—standard potassium acetate catalyst (Evonic, Warsaw, Poland)), blowing agent (n-penate from Merc, Poznan, Poland and cyclopentane from Sigma-Aldrich, Poznan, Poland) and surfactant (TEGOSTAB^®^ B 8462 from Evonic—a silicone surfactant) were placed in a plastic cup and vigorously mixed at 1500 rpm. mechanical stirrer for 60 s. Component B was diphenylmethane diisocyanate (Purocyn B from Purinova, Bydgoszcz, Poland).

### 2.2. Impregnation of SC with Rapeseed Oil (SC_O)

Press sunflower cake was milled and wetted with rapeseed oil (in the amount to cover the filler). The mixture was thoroughly mixed and poured into cups. Subsequently, the cups with the mixture were put into the vacuuming dish and the vacuuming process has proceeded until 0.01 MPa of pressure was achieved. Then, the vacuum was left for another 30 min. Ten cycles were done for the mixtures and, after that, all mixtures were thermally treated at 70 °C for 24 h. After the thermal treatment, the mixtures were left to cool down at 23 ± 5 °C temperature and 50 ± 5% humidity conditions.

### 2.3. Silanization of SC (SC_S)

Silanization was performed to improve the thermal stability of the SC and interactions with composites PUR. In order to increase the efficiency, the silanization process had to be preceded by alkalization in order to remove residual waxes and oils from the surface of SC particles.

In the first step, the SC filler was pre-treated with NaOH solution (Sigma-Aldrich, Poznan, Poland). The SC filler was soaked with NaOH solution (10%, *v*/*v*) and, after 1 h, the dispersion was neutralized with acetic acid (1%, *v*/*v*) (Sigma-Aldrich, Poznan, Poland). The SC filler was washed, deionized, and dried in an oven (80 °C) to the constant weight. In the next step, the pre-treated SC filler was subjected to silanization treatment. The obtained SC filler was soaked with a mixture of triphenylsilanol in ethanol (5%, *v*/*v*). After 3 h, the solution was removed by evaporation. SC_S filler was washed with deionized water and dried in an oven (80 °C) to constant weight.

### 2.4. Synthesis of PUR Composite Foams

PUR composite foams were produced following the method reported in the previous works [7,21,22]. In brief, the synthesis of PUR composite foams modified with the addition of SC was as follows: The previously modified SC fillers were added to the polyol premix and mixed for 60 s to obtain a homogeneous dispersion. The calculated amount of isocyanate (Purocyn B) was added to the reaction mixture and thoroughly mixed for 10 s. The free rise PUR composite foam was left at room temperature for 24 h to provide complete curing of composites. Table 1 presents the formulations of PUR composite foams containing SC filler. The final biological content in the composite was 0.7 wt %. Our research to date shows that this is the optimal content of PUR modifiers. With more natural additives, the structure of the foam changes unfavorably. A much greater heterogeneity of the structure is noticeable, with a much greater dispersion of particles, with a tendency to reduce the pore size and the presence of an open-cell structure.

### 2.5. Sample Characterization

The dynamic viscosity of polyol premixes was examined following ISO 2555:2018 [23] using Viscometer DVII+ (Brookfield, Germany). FTIR spectra were obtained using Nicolet iS50 spectrometer (Thermo Fisher Scientific, Waltham, MA, USA). The cellular structure of PUR foams and morphology of SC powders was evaluated using a scanning electron microscope using JSM-5500 LV (JEOL Ltd., Tokyo, Japan). Preparation of samples for measurement consisted of placing a double-sided self-adhesive carbon foil on special tables and gluing the tested sample to it. Then, a gold layer was applied to the sample prepared in this way using the Cressington Sputter coater 109 auto vacuum sputtering machine at a pressure of over 40 mBa, for 60 s. The sample prepared in this way was placed in a scanning electron microscope chamber (JEOL Ltd., Tokyo, Japan) and the measurement was performed.The cell sizes of PUR foams was determined by ImageJ 1.53 software [24]. (Java 1.8.0, MediaCybernetics Inc., Rockville, MD, USA). The apparent density of PUR foams was calculated as the ratio between the weight and volume of the samples according to ISO 845:2010 [25]. The number of closed-cells was evaluated according to ISO 4590:2016 standard [26]. Thermal conductivity (λ) of PUR foams was measured at 20 °C by using LaserComp 50 (TA InstrumentsInc., New Castle, DE, USA). The mechanical performances of PUR foams were performed using Zwick Z100 Testing Machine (Zwick/Roell Group, Ulm, Germany). Compressive strength (*σ*_10%_) was examined parallel to the foam rise direction according to ISO 844:2014 [27] standard. Flexural (*σ_f_*) and impact strength (*σ_I_*) of PUR foams were evaluated according to ISO 178:2019 [28] and ISO 180:2019 [29] standards (Zwick/Roell Group, Wrocław, Polska). Surface hydrophobicity of PUR foams was measured using contact angle goniometer OEC-15EC (DataPhysics Instruments GmbH, Filderstadt, Germany) with software module SCA 20. Water absorption of PU foams was performed according to ISO 2896:2001 [30].

Thermogravimetric analysis (TGA) test was performed in the function of temperature (0–600 °C) (MettlerToledo, Greifensee, Switzerland).

## 3. Results and Discussion

### 3.1. Topography and an Average Size of SC Fillers

**Figure 1:**
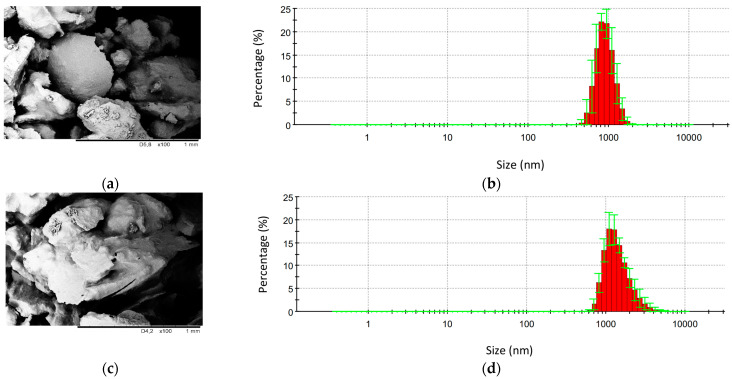

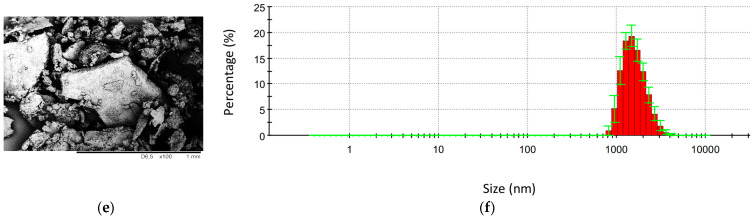
Topography and particle size distribution of (**a**,**b**) SC; (**c**,**d**) SC_S; and (**e**,**f**) SC_O.

The external morphology of SC, SC_S, and SC_O is presented in Figure 1. Comparing SC and SC_S, it is clear that the chemical treatment does not affect the topography of the filler. After the silanization, the filler possesses a similar structure; however, the particles tend to agglomerate forming bigger cluster filler particles. The size of SC particles ranges from 460 nm to 2 µm, while, after the silanization, the average size of particles increases, and it ranges from 615 nm to 1.7 µm. Similar dependence is observed for the filler impregnated with rapeseed oil, for which the average diameter ranges between 615 nm and 6.4 µm, indicating the high tendency of the filler to agglomerate in a polyol premix. Among all kinds of fillers, the SC_S has the most non-uniform morphology with many cracks. Previous studies confirmed that the chemical treatments, especially alkalization, remove the waxy substances, which are responsible for the smooth surface of the filler. Due to this, the structure of the treated fillers becomes rough, which, in turn, may result in the better interlocking of the filler in the polymeric matrix [21,22].

### 3.2. The Impact of SC Fillers on Viscosity and Processing Parameters of Polyol Premixes

Following the results presented in Table 2, the addition of SC fillers affects the viscosity of polyol premixes. When compared with PUR without the addition of any fillers, the incorporation of 3 wt % of SC fillers significantly increases the viscosity of the premixes. The greatest increase has been recorded in the case of premixes containing SC_O—the viscosity increases rapidly from 840 mPa·s to 1800 mPa·s. This result is not surprising, considering a high tendency of the filler to agglomeration and formation of coarse domains. Interestingly, among the fillers, the lowest viscosity was recorded for premixes containing unmodified SC. This, in turn, may be related to the fact that, on its surface, there are the most active cellulosic and lignin groups (e.g., hydroxyl groups), which, through interaction with polyester polyol, best disperse in polyol premixes. Similar results can also be found in earlier studies [21,22].

The foaming parameters of PUR premixes modified with SC addition were defined by measuring the processing times, which involve: cream, growth, and tack-free time. The results presented in Table 2 indicate that the addition of SC fillers significantly affects the foaming characteristics of PUR premixes. According to the results, the addition of SC, SC_S, as well as SC_O, significantly extends the cream and growth times, and this effect is the most prominent after the incorporation of SC_O. When compared with PUR, the cream time increases from 40 s to 51 s, while the growth time increases from 270 s to 350 s. The main reason for extended times may have been connected with an increased viscosity, which effectively reduces the expansion of the cells and causes the resistance to the foaming process. Moreover, due to this, the mobility of the polymer is significantly limited and the rate of the polymerization reaction is much slower. A reduced amount of CO_2_ released during the reaction between isocyanate and water affects the foaming process, leading to the prolonged expansion time of the modified PUR premixes [31,32,33,34].

### 3.3. The Impact of SC Fillers on Morphology, Apparent Density, and Thermal Conductivity of PUR Composites

**Figure 2:**
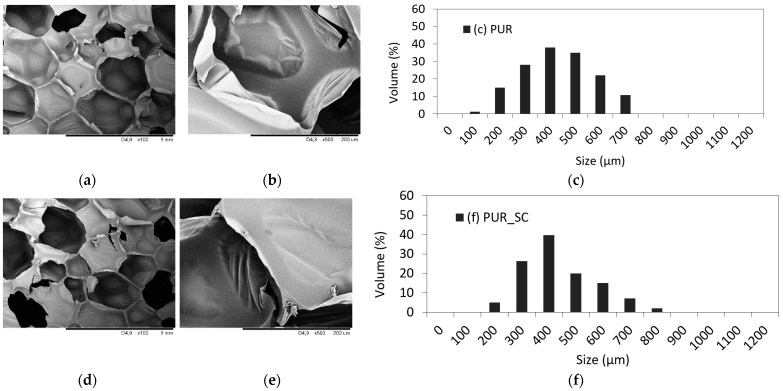

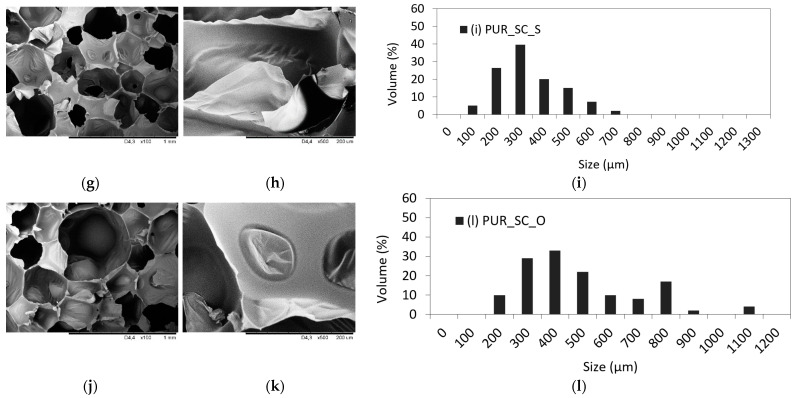
Cellular morphology and cell size distribution of (**a**–**c**) PUR; (**d**–**f**) PUR_SC; (**g**–**i**) PUR_SC_S; (**j**–**l**) PUR_SC_O.

The effect of SC, SC_S, and SC_O on the cellular morphology of PUR foams has been evaluated by SEM analysis, and the results are presented in Figure 2. The cellular structure of pristine PUR is smooth and regular. With the incorporation of SC, it becomes more heterogeneous with a greater number of open cells, which is more prominent in the case of PUR foams containing 3% of SC_O. Compared to PUR foams with the addition of non-treated SC and SC_S, the number of broken cells is increased. It is also found that PUR foams with SC_O exhibit some agglomerates of filler in the cellular structure. Such alteration in PUR morphology can be connected with increased viscosity of PUR foams with the addition of solid fillers and self-association of the SC and SC_O. Therefore, the formation and expansion of air bubbles is suppressed, which results in the formation of a more irregular structure of PUR foams. Similar dependence was found in previous studies. It was reported that the deterioration of PUR structures may be connected with the attachment of filler particles to the cell struts, which results in weakening of the PUR foam morphology and cell coalescence [7,21,22].

The average cell size and physical properties of PUR foam’s morphology are presented in Figure 2. As demonstrated in previous works, the filler particles act as nucleation sites and result in the formation of a higher number of smaller sizes. Therefore, the cell diameter decreases with the addition of non-treated and SC_S filler. When compared with PUR foams, the addition of SC and SC_S decreases the average size of cells to 415 µm and 405 µm, respectively, against 440 µm for neat PUR foam. However, an opposite tendency is observed after the incorporation of SC_O, due to the agglomeration of the particles in the polyol premixes—the cell diameter increases to 410 μm. It can be concluded that the addition of 3 wt % of filler promotes the formation of broken cells probably due to the agglomeration of the filler particles and less homogenous loading distribution in the PUR matrix. This results in the formation of clusters with greater cell diameter. The addition of non-treated and SC_S promotes the formation of smaller cells, probably due to better distribution of the filler, which enables a greater surface area for nucleation of air bubbles.

**Figure 3:**
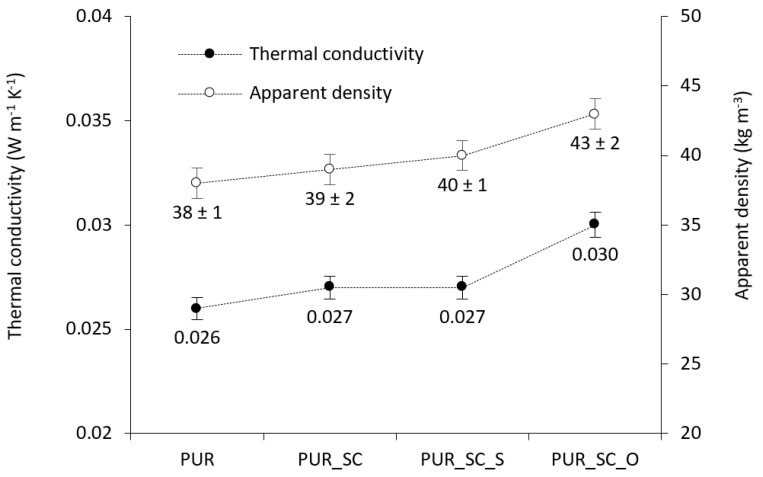
The impact of SC fillers on the apparent density and insulating properties.

This hypothesis is supported by the results of apparent density. The impact of SC fillers on the density of PUR foams is presented in Figure 3. The average density of the pristine PUR foam is 38 kg m^−3^. An increase in density is observed after the incorporation of SC. The apparent density increases to 39 kg m^−3^ for PUR foams containing 3 wt % of non-treated SC. This was probably due to the higher viscosity of PUR systems containing SC, which resulted in a slower gelation reaction with the increased SC content. A slower gelation reaction results in more gases escaping from the foam structure and hence smaller void volumes and higher densities. Similar dependence is observed in the case of PUR foams containing SC_S and SC_O—the apparent density increases to 40 kg m^−3^ and 43 kg m^−3^, respectively. Interestingly, in comparison to PUR foams containing SC_S and SC_O with the same filler content, a relatively higher density was obtained after the incorporation of SC_O. A potential reason may be found in a greater weight of the SC_O.

The differences in cellular morphology affect the thermal conductivity of PUR composite foams. Thermal conductivity of PUR composite foams was calculated via the following formula: λ = λ_s_ + λ_g_ + λ_c_ + λ_r_, where the individual parameters refer to the heat transfer coefficient of a solid phase (λ_s_), heat transfer coefficient of gas-phase (λ_g_), heat transfer coefficient connected with convection across the voids (λ_c_), and heat transfer coefficient attributed to the radiation through the voids (λ_r_) [35,36]. According to the results presented in Figure 3, the addition of SC and SC_S does not affect the insulating properties; however, the addition of SC_O increases the value of λ to 0.030 W m^−1^K^−1^, and the value still meets the requirements of National Standards established for insulating materials [37]. The deterioration of insulating properties observed for PUR composite foams should be attributed to the changes in the cellular morphology after the incorporation of SC fillers, which is observed especially in the case of composites filled with SC_O. SEM results (Figure 2) have revealed that the incorporation of SC_O results in the formation of PUR composites with a greater number of open-cells, which effectively increases the value of λ_g_ and λ_r_. According to Figure 3, the value of thermal conductivity is in line with the results of apparent density. Since the particles of SC_O possess a relatively high weight, the value of λ_s_ is higher, when compared with PUR foams without the addition of any fillers. Such explanation may be also found in the case of PUR composites filled with SC and SC_S; however, the changes in cellular morphology of the foams are not as significant as in the case of composites filled with SC_O. The deterioration of insulating properties has also been reported for PUR composite foams filled with a basalt waste—the addition of 3–40 wt % of the filler increased the value of λ to 0.025 W m^−1^K^−1^, even though the number of closed-cells has increased [9].

### 3.4. The Impact of SC Fillers on Mechanical Performances of PUR Composites

Mechanical properties of porous materials depend on several factors, such as closed-cell content and apparent density of the foams. In the case of PUR composite foams, reinforced with selected inorganic or organic fillers, the mechanical performance is influenced by the interaction between polymer matrix and filler surface.

**Figure 4:**
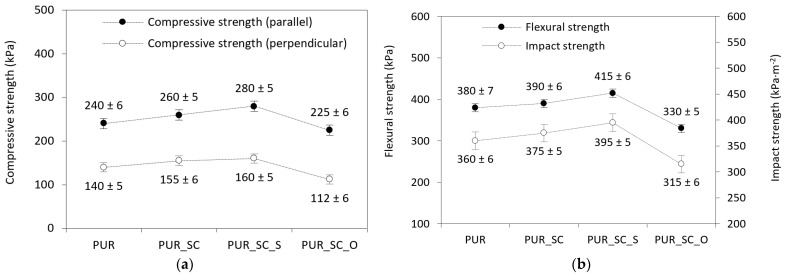
Impact of SC fillers on mechanical characteristics of PUR composites—(**a**) compressive test, (**b**) three-point bending test and impact test.

As shown in the SEM section (Figure 2), agglomeration of the filler particles occurred with the introduction of the SC_O, and there were also defects in the PUR structure. Thus, the mechanical properties of PUR modified with SC_O deteriorate due to the higher content of broken cells and the weakened structure of the resulting foams (Figure 4a). The compressive strength of PUR foams reinforced with SC_S is higher compared to PUR foams containing an impregnated filler. This is due to the fact that the filler–filler interactions, and thus the tendency of the filler particles to aggregate and agglomerate, decrease on the short side of the SC surface modification with triphenylsilyl groups, resulting in better dispersion in the foam matrix. The latter, desired effect leads to a more uniform cell structure and greater strength of the foams, thanks to which the mechanical properties of PUR foams reinforced with SC_S are improved.

The addition of SC fillers affects the value of flexural (*σ_f_*) and impact strength of PUR composite foams (Figure 4b). When compared with neat PUR, the mechanical performances have been enhanced after the incorporation of non-treated and SC_S fillers—the value of *σ_f_* increases by around 3% and 9%, while the impact strength increases by near 4% and 10%, respectively. A deterioration in mechanical performances has been reported after the addition of SC_O—the value of *σ_f_* decreases by around 13%, while the impact strength decreases by near 12%. This decrease is attributable to the large agglomeration of the impregnated particles which exhibit strong filler–filler interactions. The resulting agglomerates become weak points where stresses concentrate, resulting in faster crack propagation. Similar results are reported in previous studies. On the other hand, it has been proved that the chemical treatment of SC, such as silanization, results in the formation of PUR composites with improved mechanical performances. The rough morphology of the filler facilitates the mechanical interlocking between the polymer and filler surface and polymeric matrix, forming the restraining structure and effectively enhancing the mechanical performances of composites [38]. Improved compatibility and homogenous dispersion of the filler result in the synthesis of PUR composite foams with a uniform structure and a reduced number of empty voids. An increase in closed-cell number and decrease in cell average diameter contribute to improved mechanical characteristics because more cell walls (per unit area of the composites) can withstand the external force. A good distribution of the filler and location of the modified particles in the composite struts strengthens the PUR structure, consequently improving the mechanical characteristics.

### 3.5. The Impact of SC Fillers on Water Uptake of PUR Composite Foams

The hydrophobicity of PUR foams used as insulation materials is a very desirable parameter that determines the durability and comfort of use of porous materials. For this reason, efforts are made to reduce the wettability of foams by water. In addition to the porous structure, surface energy and the additives used are of key importance for the affinity to water, which can significantly affect water absorption and contact angle.

The introduction of an organic waste additive, which is SC sunflower cake, not only changes the structure of the foam but also slightly changes its hydrophobic character depending on the filler used. Sunflower cake, which is produced in the process of pressing sunflower oils, is always characterized by oil residue in the mass, thanks to which they have a hydrophobic character. This is manifested by an increase in the water contact angle on the surface of the foam containing the addition of unmodified SC compared to the reference foam (Table 3). As it was easy to predict, the introduction of SC impregnated foams resulted in the greatest increase in hydrophobicity, manifested both by the highest wetting angle (140°) and the lowest water sensitivity. In turn, the chemical modification of SC contributed to the reduction of hydrophobicity of sunflower cakes. Although silanization increases the hydrophobicity, the alkaline pre-treatment results in a more hydrophilic character by removing oils and waxes from the surface of the fibers. Thus, the affinity of the foams with the addition of SC_S to water was the highest among all modified foams, but still lower than that of the reference foam. The reduction of hydrophobicity was caused not only by the nature and polarity of the fillers but also by their influence on changing the structure to a more irregular one.

### 3.6. The Impact of SC Fillers on the Thermal Stability of PUR Foams

**Figure 5:**
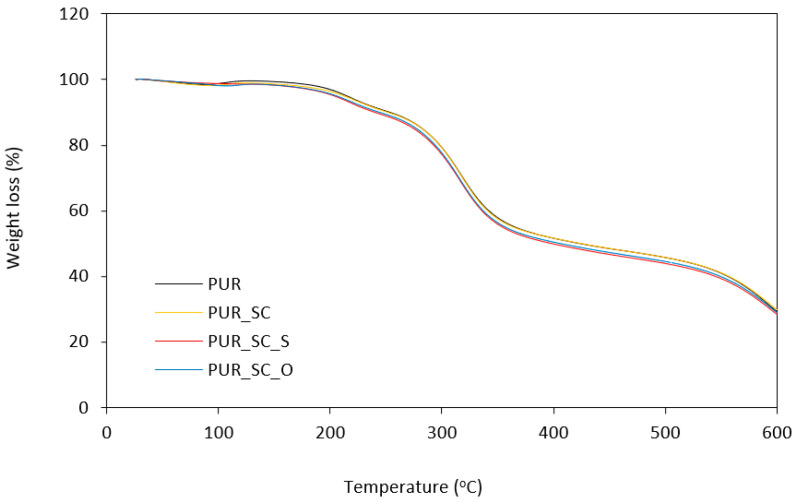
TGA curves obtained for PUR foams modified with sunflower cakes.

**Figure 6:**
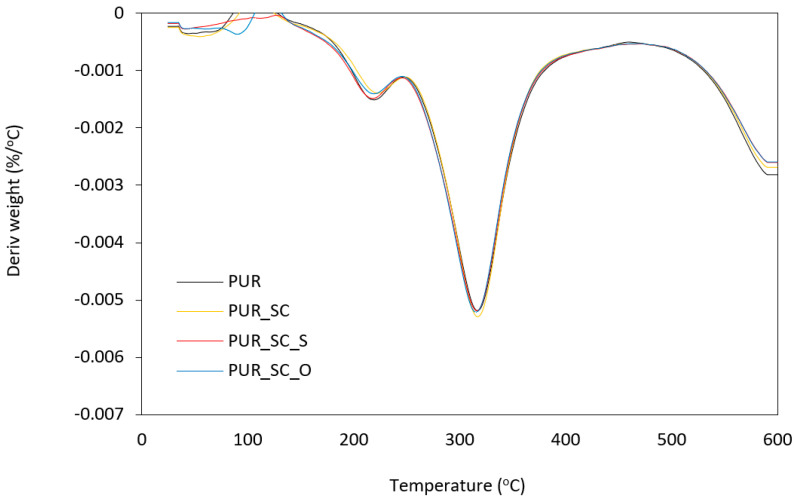
DTG curves obtained for PUR foams modified with sunflower cakes.

The thermal stability measurements are shown in Figure 5, Figure 6 and Table 4. The thermal stability was analyzed based on the temperatures at 10%, 50%, and 80% weight loss. Each foam degrades similarly. Thermal degradation of PUR takes place in three stages. The first stage of degradation seen at 150 °C to 250 °C, which corresponds to about 10% weight loss, is due to dissociation of the urethane bonds, corresponding to the degradation of hard segments [39]. In the second stage of degradation showing about 50% loss of initial weight, starting at 300–350 °C, it corresponds to the thermal decomposition of PUR soft polyol segments [40]. On the other hand, in the third stage of degradation, which manifests itself in a loss of approximately 70% of the initial weight, which occurs between 500 °C and 600 °C, the fragments formed in the previous stage are broken down into volatile products [41].

Based on Table 4, it can be seen that sunflower cake subjected to chemical modification, and in particular physical modification, has a slightly greater influence on the thermal stability of PUR foam composites than PUR_SC. This manifests itself in higher temperatures with 10%, 50%, and 80% weight loss. This may be due to the fact that solid particles embedded in the porous structure act as a thermal barrier, which significantly reduces heat transfer and slows down further degradation of PUR composite foams. In addition, this effect is supported by the more cross-linked foam structure resulting from the reaction between the filler and isocyanate functional groups. Moreover, as the filler BP content increases, the residual weight (at 600 °C) increases from 24.4% (for pure PUR) to 28.3% for PUR_SC_O composite foams.

## 4. Conclusions

The paper presents the influence of sunflower cakes as a filler. The sunflower cake was used unmodified (SC) and after chemical modification with triphenylsilanol (SC_S) and physical modification with rapeseed oil (SC_O). The research shows that the introduction of the filler significantly affects the rheological properties of the polyol premixes and the processing time. This is reflected in the formed structure of modified foams, in which the addition of fillers leads to the formation of smaller and more irregular cells, while affecting a higher density of the formed foams. Changes in the structure through the use of sunflower cakes with a different surface character also had a great impact on the insulating, mechanical, and hydrophobic properties of the foams obtained.

It was found that the addition of SC_S had the greatest impact on the improvement of mechanical properties (the compressive strength increased by approx. 15% and the impact strength by approx. 10% compared to unmodified foam) without a significant deterioration of the insulating properties. On the other hand, the introduction to the SC_O system reduced water absorption by half, which was also manifested by a much greater hydrophobicity of the foam compared to the model system.

## Figures and Tables

**Table 1:** Composition of PUR composite foams.

Component	PUR	PUR_SC	PUR_SC_S	PUR_SC_O
	Parts by Weight (wt %)			
STEPANPOL PS-2352	100	100	100	100
PUROCYN B	160	160	160	160
Kosmos 75	6	6	6	6
Kosmos 33	0.8	0.8	0.8	0.8
Tegostab B8462	2.5	2.5	2.5	2.5
Water	0.5	0.5	0.5	0.5
Pentane/cyclopentane	11	11	11	11
Sunflower cake (SC)	0	2	0	0
Silanized sunflower cake (SC_S)	0	0	2	0
Impregnated sunflower cake (SC_O)	0	0	0	2

**Table 2:** The impact of SC fillers on reological and foaming parameters of PUR premixes.

Sample	Dynamic Viscosity *η* (mPa·s)			Processing Times (s)		
	0.5 RPM	50 RPM	100 RPM	Cream Time	Growth Time	Tack-Free Time
PUR	840 ± 9	430 ± 7	320 ± 8	40 ± 2	270 ± 9	365 ± 9
PUR_SC	1200 ± 10	950 ± 9	460 ± 10	48 ± 1	315 ± 11	330 ± 9
PUR_SC_S	1350 ± 10	1000 ± 10	490 ± 12	45 ± 2	330 ± 10	335 ± 12
PUR_SC_O	1800 ± 11	1450 ± 12	650 ± 11	51 ± 2	350 ± 9	320 ± 8

**Table 3:** The impact of SC fillers on water absorption and contact angle of PUR composite foams.

Sample	Water Absorption (%)	Contact Angle (°)
PUR	20	123
PUR_SC	16	130
PUR_SC_S	17	125
PUR_SC_O	10	140

**Table 4:** The results of TGA and DTGA analysis.

Sample	T_10%_ (°C)	T_50%_ (°C)	T_80%_ (°C)	Char Residue (at 600 °C)	DTG (°C)	DTG (%/min)
PUR	209	457	585	24.4	308	0.0050
PUR_SC	206	430	581	22.3	309	0.0051
PUR_SC_S	210	459	586	22.3	311	0.0052
PUR_SC_O	213	468	588	28.3	322	0.0056

## Data Availability

The data presented in this study are available on request from the corresponding author.
